# Inhibition of FOS‐Like Antigen 1 Reduces Chemoresistance to Temozolomide Through Stemness Reprogramming via IL‐6/STAT3^Tyr705^ Pathway

**DOI:** 10.1002/mco2.70593

**Published:** 2026-01-18

**Authors:** JeongMin Sim, JeongMan Park, JinHyung Heo, Yu Jin Kim, Dongkil Kim, Yong Hyun Jeon, Jae‐Eon Lee, Seon Hee Choi, Hak Jin Kim, So Jung Hwang, Junhyung Kim, Kyung Gi Cho, Jihwan Yoo, Ju Hyung Moon, Jong‐Seok Moon, Kyoung Su Sung, Jaejoon Lim

**Affiliations:** ^1^ Department of Biomedical Science, College of Life Science CHA University Seongnam Republic of Korea; ^2^ CHA Future Medicine Research Institute CHA Bundang Medical Center Seongnam Republic of Korea; ^3^ Department of Medicine, College of Medicine Hallym University Chuncheon Republic of Korea; ^4^ Department of Pathology, Bundang CHA Medical Center CHA University College of Medicine Seongnam Republic of Korea; ^5^ Preclinical Research Center (PRC) Daegu‐Gyeonbuk Medical Innovation Foundation (K‐Medi Hub) Daegu Republic of Korea; ^6^ Department of Radiology Pusan National University Hospital Biomedical Institute of Pusan National University Hospital Pusan National University School of Medicine Busan Republic of Korea; ^7^ Department of Neurosurgery Bundang CHA Medical Center CHA University College of Medicine Seongnam Republic of Korea; ^8^ Development Division CG Cell Corporation Seongnam Republic of Korea; ^9^ Department of Integrated Biomedical Science Soonchunhyang Institute of Medi‐bio Science (SIMS) Soonchunhyang University Cheonan Republic of Korea; ^10^ Department of Biology Education Kongju National University Gongju Republic of Korea; ^11^ Department of Neurosurgery Brain Tumor Center Gangnam Severance Hospital Seoul Republic of Korea; ^12^ Department of Neurosurgery Endoscopic Skull Base Center Severance Hospital Yonsei University College of Medicine Seoul Republic of Korea; ^13^ Department of Pathology College of Medicine Soonchunhyang University Cheonan Republic of Korea; ^14^ Department of Neurosurgery Dong‐A University Hospital, Dong‐A University College of Medicine Busan Republic of Korea

**Keywords:** glioblastoma stemness, glioblastoma, FOSL1, IL‐6 signaling pathway, TMZ chemoresistance

## Abstract

Glioblastoma (GBM) is the most lethal brain tumor, characterized by strong resistance to conventional therapies. Despite recent therapeutic advancements, overcoming chemoresistance remains a major challenge. Here, we identified FOS‐like antigen 1 (FOSL1) as a novel therapeutic target in GBM, particularly in patients with resistance to conventional drugs, including temozolomide (TMZ). FOSL1 gene was identified from the DepMap database as a potential mediator of TMZ resistance in GBM and found to be associated with chemoresistance molecular signatures and poor clinical outcomes. Functional analyses in GBM cells revealed that FOSL1 suppression enhanced apoptosis, induced G0/G1 cell cycle arrest, and reduced both cell migration and stemness marker expression. Transcriptomic profiling, including single‐cell RNA‐seq and bulk RNA‐seq, highlighted the pivotal role of the interleukin‐6 (IL‐6)/STAT3 signaling pathway in FOSL1‐mediated stemness. Mechanistically, in vitro experiments demonstrated that FOSL1 induces GBM stemness through IL‐6‐pSTAT3^Tyr705^ signaling axis. Furthermore, vemurafenib, which targets FOSL1, was identified as a potential therapeutic agent against TMZ‐resistant GBM in a mouse model. These findings suggest that FOSL1 promotes TMZ chemoresistance by regulating IL‐6‐pSTAT3^Tyr705^‐mediated stemness in GBM cells, making it a promising therapeutic target to overcome chemoresistance in GBM.

## Graphical abstract

The role of FOSL1 in TMZ response to GBM. FOSL1 enhances TMZ resistance by promoting cell cycle progression, MGMT expression, and IL‐6‐pSTAT3^Tyr705^‐stemness axis. VEM is a potential candidate drug for alleviating TMZ resistance in GBM, highlighting a new approach for chemo‐resistant GBM treatment.

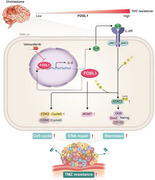

## 1 Introduction

Glioblastoma (GBM) is among the most incurable brain tumors (median survival < 15 months), with a high likelihood of recurrence [1]. However, no major progress has been made since the application of temozolomide (TMZ) to GBM, despite improvements in diagnostic modalities and comprehensive therapeutic approaches [2]. TMZ is the gold standard chemotherapy for primary or recurrent GBM because of its ability to overcome the restrictive blood–brain barrier (BBB), fewer adverse effects, and efficiency in extending patient lifespan [3]. Typically, an aggressive conventional treatment schedule involves the administration of TMZ alkylating chemotherapy (six cycles for 5 days every month) concomitantly with ionizing radiotherapy (daily); however, its efficacy remains limited [4]. Furthermore, GBM exhibits high drug resistance, which poses a significant challenge for improving treatment efficacy and reducing relapse rates. Several studies on chemoradiotherapy in GBM treatment have demonstrated that chemoresistance to TMZ is a challenge for improving outcomes [5, 6]. Therefore, promising approaches to counteract chemoresistance and enhance the effectiveness of GBM treatments are urgently required.

The mode of action of TMZ in patients with GBM mostly involves alkylation of O^6^‐guanine, N^3^‐guanine, N^7^‐guanine, and N^3^‐adenine. This alkylation promotes aberrant DNA base pairing, resulting in genomic instability and ultimately triggering apoptosis in tumor cells [7]. However, DNA repair systems such as O‐6‐methylguanine DNA methyltransferase (MGMT) and base excision repair (BER) can mitigate TMZ‐induced single‐ and double‐stranded DNA breaks, contributing to TMZ chemoresistance [8, 9]. In addition to DNA repair mechanisms, several other molecular pathways and processes have been implicated in TMZ resistance. These include PI3K/Akt, Wnt/β‐catenin, JAK/STAT, autophagy, epigenetic modifications, and microRNA inhibition of TMZ responsivity [10, 11, 12]. Multiple contributors to TMZ chemoresistance have been identified; however, the involvement of specific transcription factors in this process remains to be fully elucidated.

FOS‐like antigen 1 (FOSL1; gene name: *FRA1*) is a subunit of the transcriptional complex AP1, which functions through dimerization with JUN family proteins and regulates genes involved in cell proliferation, differentiation, and survival [13]. Emerging evidence indicates that FOSL1 is upregulated in GBM and facilitates tumor progression by modulating several oncogenic pathways, including MAPK, PI3K/Akt, TGF‐β, and Wnt/β‐catenin [14, 15, 16]. FOSL1 also promotes chemotherapy resistance in various types of tumors, including gliomas, colon cancer, ovarian cancer, and breast cancer [17, 18, 19, 20]. The mechanisms underlying FOSL1‐induced chemoresistance in solid tumors include reprogramming of stemness, activation of EMT, interleukin‐6 (IL‐6)/STAT3, ERK/JNK, and Wnt/β‐catenin signaling pathways [16]. Despite a clear understanding of the role of FOSL1 in chemoresistance in multiple cancer types, its specific resistance to TMZ, the commonly used drug for GBM treatment, remains unclear. Therefore, it is worth considering the potential contribution of FOSL1 to the receptive capacity of TMZ in patients with GBM.

Tumorigenic GBM stemness signatures, including CD133, OLIG2, CD44, SOX2, Oct4, and Nanog, have been shown to induce TMZ chemoresistance in GBM [21, 22, 23, 24, 25]. Glioma stem cells (GSCs), while constituting a minor subpopulation within the tumor mass, are largely responsible for chemoresistance and tumor recurrence due to their intrinsic plasticity and their capacity to promote intratumoral heterogeneity [26]. Previous studies have identified several FOSL1‐mediated stemness inducers such as IL‐6/STAT3 and SNAI3 in breast cancer, suggesting that drug resistance is induced by the FOSL1‐IL‐6/STAT3‐stemness axis [27, 28]. However, the specific role of this axis in TMZ‐resistant GBM remains unclear.

In this study, we aimed to investigate the function of the FOSL1‐IL‐6/STAT3‐stemness axis in TMZ chemoresistance and explore the potential of vemurafenib (VEM) as an adjuvant for TMZ.

## 2 Results

### 2.1 FOSL1 is Associated With TMZ Resistance and Poor Prognosis in Patients With GBM

**FIGURE 1:**
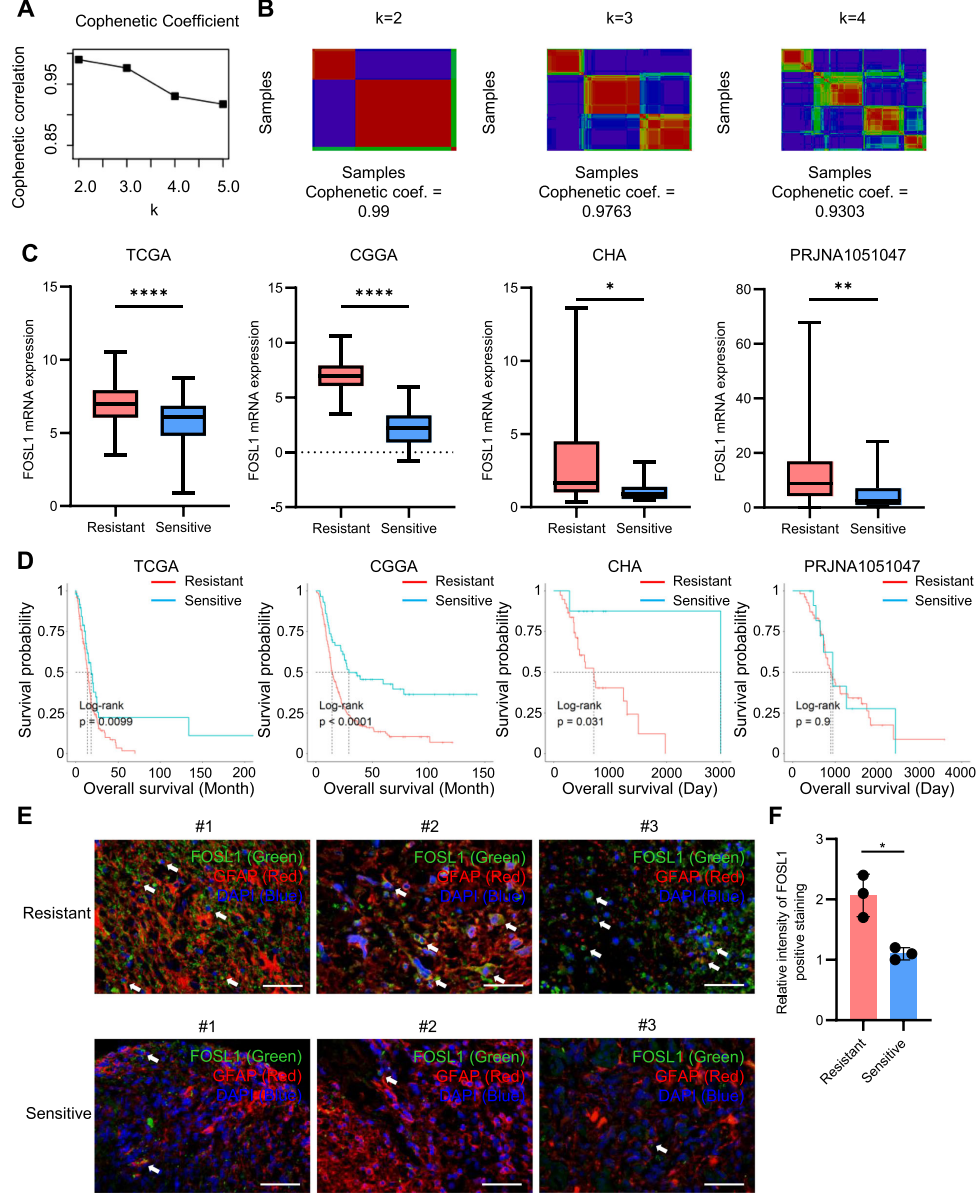
FOSL1 is associated with chemoresistance and poor prognosis in patients with GBM. (A, B) Cophenetic coefficients between NMF clustering with the expression of 146 genes positively correlated with TMZ resistance in the DepMap dataset. (C) Comparison of expression of *FOSL1* between the two groups in four datasets. (D) Results of survival analysis between groups divided by NMF clustering in the four datasets. E Representative immunofluorescence images of FOSL1 and GFAP staining in patients with TMZ resistant GBM (Resistant #1, #2, and #3, *n* = 3) or TMZ resistant GBM (Resistant #1, #2, and #3, *n* = 3) showing FOSL1 (green), GFAP (red), and DAPI‐stained nucleus (blue). Scale bars, 50 µm. White arrows indicate FOSL1 and GFAP‐positive tumor cells. (F) Quantification for intensity for FOSL1‐positive staining in tumor cells from immunofluorescence images of (E). Data are presented as mean ± SD. *p < 0.05; **p < 0.005; ****p < 0.00005; paired *t*‐test.

To investigate the molecular determinants contributing to TMZ resistance, we conducted correlation analysis in Dependency Map (DepMap) portal database, revealing that significantly positively correlated genes were associated with FOSL1, the regulation of the cell cycle, and mitotic division, according to the results of the gene set enrichment analysis (GSEA; Figure S1A,B). Moreover, the expression levels of *FOSL1* gene was elevated in mesenchymal subtypes across four cohorts including The Cancer Genome Atlas (TCGA), Chinese Glioma Genome Atlas (CGGA), CHA, and PRJNA1051047 (Figure S1C). To further investigate the clinical relevance of FOSL1, non‐negative matrix factorization (NMF) clustering was performed using transcriptomic profiles of 146 genes in TCGA cohort and isocitrate dehydrogenase (IDH) wild‐type patient samples were divided into two classes (Figure 1A,B). Applying the Bayesian compound covariate method, we subsequently classified samples from the CGGA, CHA, and PRJNA1051047 cohorts into the same two subgroups. Following the classification in each cohort, we analyzed the mRNA expression of *FOSL1*, the proportion of the mesenchymal expression subtype of GBM, and the prognosis associated with the two groups. Comparative analyses revealed that the TMZ‐resistant subgroup exhibited significantly higher FOSL1 mRNA expression, an increased proportion of mesenchymal subtype tumors, and worse overall survival compared to the TMZ‐sensitive group (Figure 1C,D and Figure S1D). To validate these findings at the protein level, we evaluated FOSL1 expression in glial fibrillary acidic protein (GFAP)‐positive tumor cells—used as a GBM marker—within brain tumor tissues from patients clinically categorized as TMZ resistant or TMZ sensitive. Immunohistochemical analysis revealed that FOSL1 staining intensity was markedly elevated in tumor cells from TMZ‐resistant GBM patients relative to those from TMZ‐sensitive patients (Figure 1E). Quantitative assessment confirmed a statistically significant increase in FOSL1 protein levels in TMZ‐resistant GBM (Figure 1E,F). These results suggest that the elevated expression of FOSL1 is closely linked to TMZ chemoresistance and is indicative of poor clinical outcomes in patients with GBM.

### 2.2 FOSL1 Contributes to the Resistance in Response to TMZ in GBM Cells

**FIGURE 2:**
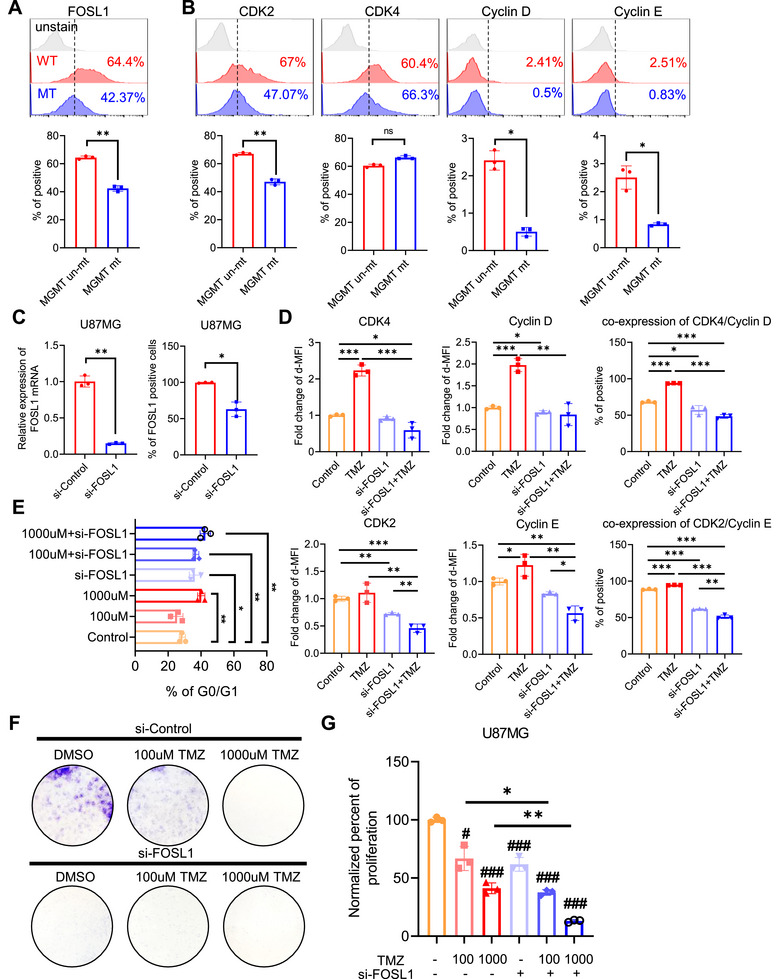
Inhibition of FOSL1 in GBM cells reverses TMZ response. (A, B) Representative histogram of protein expression, including FOSL1 and cell cycle‐associated molecules, in GBM patient's cells was shown (top panel). Protein expression in patients with GBM‐derived cells (*n* = 6), including FOSL1 and cell cycle‐associated molecules, was analyzed by flow cytometry and quantified using FlowJo V10 (bottom panel). (C) *FOSL1* mRNA expression (left panel) in U87MG treated with si‐control or si‐FOSL1 analyzed using qPCR. *GAPDH* was used as a control gene for relative quantification. FOSL1 protein expression in U87MG treated with si‐control or si‐FOSL1 was analyzed by flow cytometry and quantified using FlowJo V10 (right panel). (D) Expression of G0/G1 to S phase transition‐related proteins, including CDK4, cyclin D, CDK2, and cyclin E, analyzed using flow cytometry and quantified using FlowJo V10. E Population of the G0/G1 phase of U87MG cells stained with CCS1 and analyzed using flow cytometry. Data were quantified using FlowJo V10. (F) Representative images of colony formation showing viable U87MG cells treated with si‐control, si‐FOSL1 or TMZ (purple). (G) Proliferation of U87MG cells calculated using the WST‐8 reduction assay following the manufacturer's instructions. *p < 0.05; **p < 0.005; ***p < 0.0005; paired *t*‐test (*n* = 3).

To elucidate the role of FOSL1 in response to TMZ, a DNA alkylating drug that induces cell cycle arrest in GBM cells, we analyzed the levels of FOSL1 and G0/G1 to S phase transition‐associated proteins, including CDK2, CDK4, cyclin D, and cyclin E^29^, in primary GBM cells with unmethylated (TMZ‐resistant group) or methylated MGMT (TMZ non‐resistant group). The number of FOSL1‐positive GBM cells was higher in the MGMT‐unmethylated group than that in the MGMT‐methylated group (Figure 2A). Similarly, protein expression level of CDK2, cyclin D, and cyclin E was elevated in the unmethylated MGMT group relative to that in the methylated MGMT group (Figure 2B). Next, we examined whether *FOSL1* knockdown induced resistance to TMZ via cell cycle arrest during the G0/G1 to S phase transition. U87MG cells had higher *FOSL1* expression than the other GBM cells, so it was utilized (Figure S2A). Knockdown of *FOSL1* via siRNA significantly decreased FOSL1 mRNA and protein expression (Figure 2C). *FOSL1* knockdown suppressed the levels of CDK2, CDK4, cyclin D, and cyclin E in response to TMZ relative to those in the control (Figure 2D and Figure S3A). Moreover, *FOSL1* knockdown induced greater cell cycle arrest in the G0/G1 phase in response to TMZ treatment than that in the control (Figure 2E). Consistent with cell cycle analysis, *FOSL1* knockdown reduced the colony formation and proliferation ability of U87MG cells (Figure 2F,G). These results suggest that FOSL1 enhances resistance to TMZ in GBM cells by sustaining G1–S phase transition and supporting proliferative potential.

### 2.3 FOSL1 Contributes to the Stemness Reprogramming in GBM Cells

**FIGURE 3:**
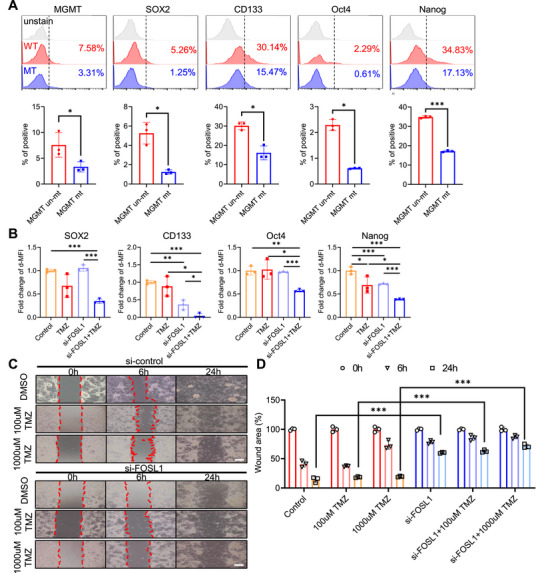
Inhibition of FOSL1 in GBM cells reduces the stemness hallmark. (A) Representative histogram of protein expression, including MGMT and stemness hallmarks, in GBM patient's cells was shown (top panel). Protein expression in patients with GBM‐derived cells (*n* = 6), including MGMT and stemness hallmarks, analyzed using flow cytometry and quantified using FlowJo V10 (bottom panel). (B) Expression of stemness‐related proteins in U87MG cells analyzed using flow cytometry and quantified using FlowJo V10. (C) Representative images of the wound healing analysis of U87MG cells treated with si‐control, si‐FOSL1, or TMZ (0, 6, and 24 h). Scale bars, 200 µm. (D) Quantification of the cell wound area at each time point in U87MG cells was conducted with ImageJ. *p < 0.05; **p < 0.005; ***p < 0.0005; paired *t*‐test (*n* = 3).

Cellular reprogramming toward a stem‐like state is a well‐documented mechanism contributing to TMZ resistance in glioblastoma [17, 30, 31]. To determine whether FOSL1 plays a role in this process, we assessed the expression of key stemness‐associated markers, including SOX2, CD133, OCT4, and NANOG, in primary GBM cells stratified by MGMT promoter methylation status. The expression levels of SOX2, CD133, OCT4, and NANOG were significantly upregulated in MGMT‐unmethylated (TMZ‐resistant) samples relative to MGMT‐methylated (TMZ‐sensitive) samples (Figure 3A). Furthermore, *FOSL1* knockdown suppressed the levels of GBM stemness molecules, including SOX2, CD133, OCT4, and NANOG, in response to TMZ compared to the control (Figure 3B and Figure S3B). Given that enhanced migratory ability is a hallmark of GBM stem‐like phenotypes [32], we examined whether *FOSL1* knockdown affected cell migration in response to TMZ. *FOSL1* knockdown significantly reduced cell migration in response to TMZ compared to that in control cells (Figure 3C,D). These results suggested that FOSL1 contributes to stemness reprogramming in GBM cells.

### 2.4 FOSL1 is Associated With IL‐6‐JAK‐STAT3 Pathway‐Related Stemness in GBM

**FIGURE 4:**
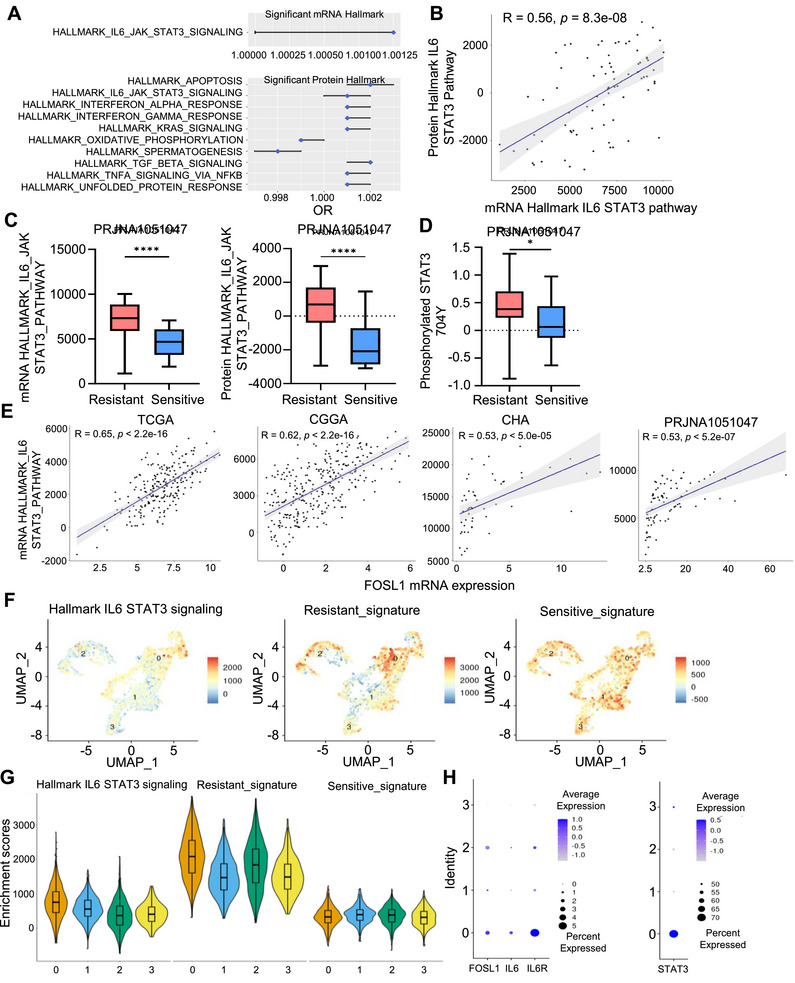
IL‐6 signaling pathway is associated with FOSL1 expression in GBM cells. (A) Results of significant logistic regression analysis with groups divided by NMF clustering and ssGSEA of RNA‐seq and global protein data. (B) Correlation between mRNA and protein IL‐6‐JAK‐STAT3 signaling pathway ssGSEA scores. (C) Comparison of mRNA and protein ssGSEA scores of the IL6‐JAK‐STAT3 signaling pathway in the Hallmark database between groups. (D) Comparison of phosphorylated 704Y STAT3 levels between groups. (E) The correlation between *FOSL1* mRNA expression and ssGSEA score based on RNA‐seq data in four cohort. (F) Dim plot of single‐cell GSEA (scGSEA) of IL‐6‐JAK‐STAT3 signaling in Hallmark, resistant, and sensitive signature. (G) Comparison of scGSEA score between four clusters of aneuploid cells. H Expression of FOSL1, IL‐6, IL6R, and STAT3 in aneuploid cells *p < 0.05; **p < 0.005; ***p < 0.0005; ****p < 0.00005.

To compare the characteristics of biological meaning between the TMZ resistant group and the TMZ sensitive groups, single sample GSEA (ssGSEA) was conducted based on transcriptomic and proteomic omics data from the hallmark‐curated database. The IL‐6‐JAK‐STAT3 was significantly associated with the TMZ‐resistant group transcriptomics and proteomics, and its score was significantly higher than that of the TMZ‐sensitive group (Figure 4A,B). Moreover, the ssGSEA score of the IL‐6‐JAK‐STAT3 pathway based on transcriptomic and proteomic data in paired samples showed a moderate positive correlation (Figure 4C). Because the phosphorylation of Y704 or Y705 in STAT3 is important for the activation of the IL‐6‐JAK‐STAT3 pathway, we compared the levels of phosphorylated STAT3 Y704, which revealed significantly higher levels in the TMZ‐resistant group than those in the TMZ‐sensitive group (Figure 4D). Finally, we confirmed a significant moderate positive correlation between *FOSL1* mRNA expression and the IL‐6‐JAK‐STAT3 ssGSEA score in the four cohorts used in this study (Figure 4E). Next, we investigated the association between FOSL1 and the IL‐6‐JAK‐STAT3 pathway in single cells. Approximately 20 K cells were classified as aneuploid and diploid, followed by annotation of well‐known cell type markers (Figure S4A). After isolating only the malignant tumor cells, we analyzed IL‐6/STAT3 signaling and the resistant and sensitive signatures of TMZ extracted from analyzing Depmap data in four clusters. The results of single‐cell GSEA showed that Cluster 0 exhibited higher IL‐6/STAT3 signaling and resistant signature scores compared to other clusters, in contrast of the sensitive signature, which did not show differences between clusters (Figure 4F,G). Additionally, higher expression levels of FOSL1, IL‐6, IL6R, and STAT3 were observed in Cluster 0 (Figure 4H). FOSL expression was higher in the (MES) aneuploid cells than in the other three types of GBM cells (Figure S4B). In addition, the ssGSEA score of aneuploid cells was compared among the four types; MES‐like aneuploid cells showed significantly higher scores than the others, similar to the results for FOSL1 expression (Figure S4C,D). These results suggest that FOSL1 is associated with IL6‐JAK‐STAT3 pathway–related stemness in GBM.

### 2.5 *FOSL1* Knockdown Reduces the Stemness Reprogramming by Inhibition of IL‐6 Signaling Pathway in GBM Cells

**FIGURE 5:**
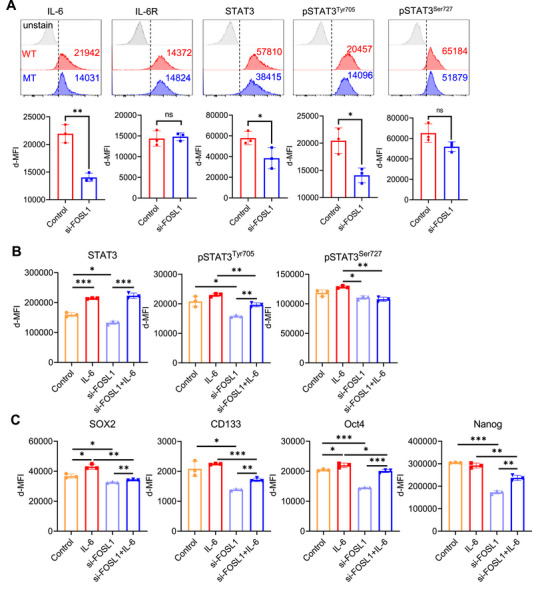
FOSL1 regulates the IL‐6/STAT3 signaling pathway, leading to GBM stemness. (A) Representative histogram for flow cytometry analysis of the IL‐6/STAT3 axis in U87MG cells (gray: unstained, red: si‐control, blue: si‐FOSL1, top panel). δ‐MFI values were shown on the histogram. Quantification of IL‐6/STAT3 signaling pathway‐associated molecules measured using flow cytometry in U87MG cells (bottom panel). (B) Quantification of IL‐6/STAT3 signaling pathway‐associated molecules measured using flow cytometry in U87MG cells. The data are shown as δ‐MFI values. (C) Quantification of stemness hallmarks measured using flow cytometry in U87MG cells. *p < 0.05; **p < 0.005; ***p < 0.0005; paired *t*‐test (*n* = 3).

To determine whether FOSL1 modulates IL‐6–dependent signaling pathways in GBM cells, we evaluated the impact of FOSL1 knockdown on key components of the IL‐6–STAT3 axis. Suppression of FOSL1 led to a marked reduction in the protein expression of IL‐6 and STAT3, as well as a decrease in the phosphorylation of STAT3 at Tyr705 (Figure 5A). Upon exogenous IL‐6 stimulation, STAT3, pSTAT3^Tyr705^, and pSTAT3^Ser727^ significantly diminished in FOSL1‐deficient cells compared to controls (Figure 5B and Figure S3C). Moreover, *FOSL1* knockdown significantly reduced the levels of stemness molecules, including SOX2, CD133, OCT4, and NANOG, in response to IL‐6 stimulation compared to the control (Figure 5C and Figure S3D). These results suggest that *FOSL1* knockdown reduces stemness reprogramming via IL‐6 stimulation in GBM cells.

### 2.6 Pharmacological Inhibition of FOSL1 Using VEM Contributes to Reduction of Chemoresistance in Response to TMZ

**FIGURE 6:**
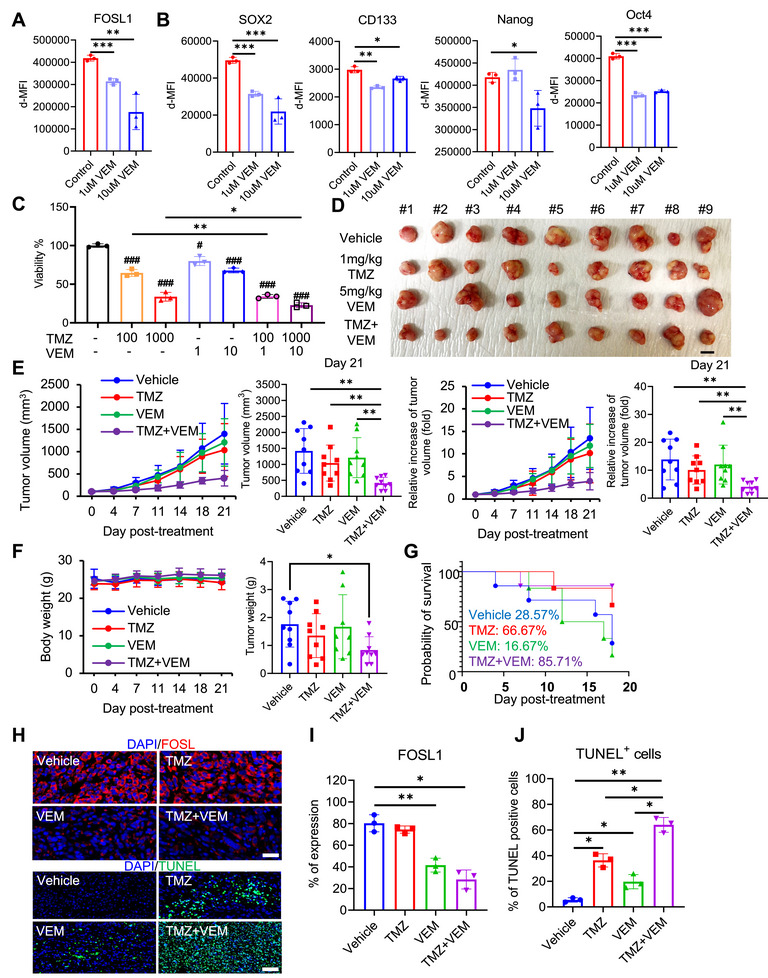
Vemurafenib sensitizes TMZ responsiveness via FOSL1 downregulation in GBM cells. (A, B) Each protein, including FOSL1 and stemness hallmark, was analyzed using flow cytometry, and δ‐MFI of proteins was quantified using FlowJo V10. C Viability of U87MG treated with TMZ or VEM was quantified by WST‐8 reduction analysis following the manufacturer's instruction. (D) Image of tumor specimens from xenograft BALB/c nude mice model. Scale bar, 10 mm. (E) Measurement of tumor volume and relative tumor volume in xenograft GBM mice for 21 days. Tumor volume (mm^3^) was measured with calipers at the indicated time points. Tumor volume (mm^3^) = *d* ^2^ × *D*/2, where *d* and *D* are the shortest and longest diameters (mm). (F) Measurement of mouse and tumor weight. (G) Kaplan–Meier curve for the orthotopic GBM mouse model treated with TMZ or VEM. (H) Representative image of immunofluorescence for FOSL1 and TUNEL expression in GBM tissues of orthotopic nude mice (blue: DAPI, red: FOSL1, green: TUNEL). Scale bars, 50 µm. (I) Expression of FOSL1 in orthotopic GBM mouse tissue quantified using ImageJ. (J) Quantification of TUNEL staining in orthotopic GBM mouse tissues. The stained cells were quantified using ImageJ. *p < 0.05; **p < 0.005; ***p < 0.0005; paired *t*‐test (xenograft model, *n* = 36; orthotopic model, *n* = 28).

To evaluate the therapeutic potential of pharmacologically targeting FOSL1 in the context of TMZ resistance, we assessed the effect of VEM, a known suppressor of FOSL1 expression in several solid tumors [33, 34]. Initial experiments confirmed that VEM treatment resulted in a dose‐dependent downregulation of FOSL1 protein levels in GBM cells (Figure 6A and Figure S3E). Given the established role of FOSL1 in regulating stemness‐associated molecules (Figure 3), we analyzed whether FOSL1 inhibition by VEM could suppress the expression of stemness molecules in GBM cells (Figure 6B and Figure S3E). VEM significantly decreased the expression of stemness molecules, including SOX2, CD133, OCT4, and NANOG, relative to the control (Figure 6B). We then investigated whether VEM could sensitize GBM cells to TMZ. Cell viability assays revealed that VEM monotherapy modestly reduced GBM cell survival, while co‐treatment with TMZ and VEM produced a significantly greater reduction in cell viability compared to TMZ alone (Figure 6C).

To validate these findings in vivo, we employed both subcutaneous and orthotopic xenograft GBM models. In the subcutaneous model, combined TMZ and VEM treatment significantly suppressed tumor growth compared to vehicle controls by day 21 (Figure 6D,E). No significant changes in the body weight were observed, but tumor weight was significantly decreased in dual treatment group compared with the control (Figure 6F). Although the group treated with VEM alone showed a tendency to have reduced tumor size compared to the control group, the difference was not significant. To facilitate clinical approaches, an orthotopic mouse model was established. In the orthotopic model, survival analysis demonstrated that the TMZ+VEM group had the most prolonged survival (Figure 6G). Histological evaluation supported these observations. Immunohistochemical staining revealed a notable decrease in FOSL1 expression in tumors treated with VEM or the TMZ+VEM combination (Figure 6H,I). Furthermore, TUNEL assays indicated an increase in apoptotic tumor cells, predominantly in the combination treatment group (Figure 6H,J). Collectively, these results suggest that the pharmacological inhibition of FOSL1 by VEM contributes to the reduction of chemoresistance in response to TMZ.

## 3 Discussion

This study elucidates the role of FOSL1 in promoting TMZ chemoresistance in GBM and its involvement in the FOSL1‐IL‐6‐STAT3^Tyr705^‐GBM stemness axis. By employing a comprehensive methodology that integrates in silico, in vitro, and in vivo studies, we identified FOSL1 as a potential therapeutic target for GBM treatment. Analysis of the DepMap data of glioma cell lines revealed a correlation between elevated *FOSL1* expression and unfavorable TMZ response in GBM, which aligns with recent findings that highlight FOSL1 as a transcription factor involved in oncogenesis and chemoresistance in breast cancer and melanoma [19, 35]. Furthermore, high *FOSL1* expression was associated with poor patient prognosis and the mesenchymal subtype across both in‐house and public GBM datasets.

Knockdown experiments further confirmed the direct involvement of FOSL1 in TMZ chemoresistance in GBM cells. Our results demonstrate that inhibiting *FOSL1* sensitizes GBM cells to TMZ treatment and reverses chemoresistance by disrupting cell cycle progression and modulating protein expression and cell proliferation. Additionally, we found that FOSL1 promotes stemness in GBM cells, thereby enhancing the survival of a subpopulation of stem‐like tumor cells known to exhibit greater resistance to standard therapies, including TMZ [31]. This indicates that FOSL1‐mediated stemness is a critical mechanism underlying TMZ resistance in GBM.

Our study suggests a potential association between FOSL1 and MGMT expression in GBM. As shown in Figure 3A and Figure S2B, we observed parallel expression patterns of FOSL1 and MGMT in patients with GBM and a decrease in MGMT levels following FOSL1 inhibition in GBM cells. While no prior reports have established a direct link between these two proteins, our findings imply that *FOSL1* may function as an upstream regulator of MGMT. Previous studies and our results have suggested that the MGMT signaling pathway regulated by FOSL1 is potentially associated with stemness hallmarks such as Nanog regulated by IL‐6 signaling pathway, and the regulatory mechanisms between them need to be elucidated [36, 37]. Meanwhile, as shown in Figure S2C,D, we also confirmed that *FOSL1* inhibition leads to downregulation of intracellular reactive oxygen species (iROS). ROS are known to play a paradoxical role in tumor cell chemoresistance, often contributing positively to TMZ response [38]. Future studies are necessary to elucidate the functions of reduced ROS following *FOSL1* inhibition and to explore the interplay between various chemoresistance mechanisms.

Another intriguing aspect of our findings is the involvement of the IL‐6 signaling pathway in the FOSL1 axis within GBM cells. Previous reports have shown that increased FOSL1 activates the IL‐6‐STAT3 signaling pathway either directly or through NLRP3‐mediated inflammation in breast cancer and psoriasis [39, 40]. Actually, single‐cell and proteomic analyses have indicated that FOSL1 is associated with stemness through the IL‐6 pathway in glioblastoma. IL‐6 is well established as a mediator of inflammatory signaling and a promoter of stem cell‐like properties in multiple cancers, including GBM [41, 42, 43]. Our results corroborate these observations, suggesting that *FOSL1* acts as a key regulator of IL‐6 signaling, thereby enhancing GBM stemness and facilitating the emergence of chemoresistance. This positions FOSL1 as an upstream regulator of IL‐6 signaling, which contributes to the persistence of therapy‐resistant tumor populations.

We also demonstrated that VEM, a FOSL1 expression inhibitor, could serve as an effective adjuvant to TMZ therapy. Our in vitro and in vivo data indicated that the combination of VEM and TMZ significantly reduced tumor growth and enhanced chemosensitivity. Although VEM is primarily used to target BRAF mutations, its implications may extend to tumors reliant on AP‐1 family transcription factors, such as *FOSL1*. The potential of VEM in combination with TMZ underscores its promise as a novel therapeutic option for patients with GBM who exhibit resistance to standard chemotherapy. Although BRAF V600E‐positive GBMs account for only 3% of whole cases, the BRAF‐FOSL1 axis appears to be potentially relevant, given that BRAF is the upstream signaling molecule of FOSL1 in colon cancer and that both molecules share the MAPK signaling pathway [33, 44]. Future investigations should further explore the functional relationship between BRAF mutation and *FOSL1* expression to fully elucidate the therapeutic mechanisms of VEM.

## 4 Conclusion

In summary, our study identifies FOSL1 as a crucial mediator of TMZ resistance in GBM through the upregulation of the IL‐6‐STAT3^Tyr705^‐stemness axis. Targeting *FOSL1* directly or through its regulatory pathways presents a promising strategy to overcome chemoresistance and enhance patient outcomes. Further exploration of *FOSL1* inhibitors, such as VEM, in clinical settings may pave the way for innovative therapeutic interventions for GBM. These findings underscore the importance of targeting the FOSL1‐IL‐6‐STAT3^Tyr705^‐stemness axis as a means to combat chemoresistance and GBM stemness, thereby providing a promising therapeutic strategy to improve outcomes for patients with GBM.

## 5 Materials and Methods

### 5.1 Human Subject Study

This study involving human participants was conducted in compliance with the principles outlined in the Declaration of Helsinki. The Bundang CHA Medical Center's Institutional Review Board examined and approved the human subject study (IRB No. CHAMC2021‐01‐024). Prior to participation, all individuals gave written informed consent. The study cohort consisted of six GBM patients: three exhibiting resistance to TMZ and three with TMZ sensitivity, as summarized in Table S1.

### 5.2 GBM Cell Lines

GBM cell lines, including U87MG, A172, U118MG, LN18, T98G, LN229, and SW1783, were sourced from the American Type Culture Collection (ATCC, Manassas, VA, USA) and were verified to be free of mycoplasma contamination. Cells were cultured in Dulbecco's modified Eagle's medium (DMEM; Thermo Fisher Scientific, Waltham, MA, USA) containing 12.5% heat‐inactivated fetal bovine serum (FBS; <5 EU/mL endotoxin; Thermo Fisher Scientific) and 1% penicillin‐streptomycin (Thermo Fisher Scientific). All cultures were maintained at 37°C in a humidified incubator with 5% CO_2_.

For treatment experiments, cells were exposed to TMZ (Millipore Sigma, Burlington, MA, USA) at concentrations ranging from 10 to 1000 µM, VEM (Selleck Chemicals LLC, Houston, TX, USA) at 1–10 µM, or recombinant human IL‐6 (Abcam, Cambridge, UK) at 10 ng/mL. All reagents and culture media were certified endotoxin‐ and LPS‐free.

Firefly luciferase‐expressing U87MG cells (U87MG/Luc2; ATCC #HTB‐14‐LUC2) were also utilized and cultured in RPMI‐1640 medium supplemented with 10% FBS, 1% penicillin‐streptomycin, and 8 µg/mL blasticidin (all from Thermo Fisher Scientific). These cells were maintained under the same incubation conditions (37°C, 5% CO_2_). Monthly mycoplasma screening was carried out using the BioMycoX PCR Detection Kit (CellSafe, Yongin, South Korea).

### 5.3 Patients With GBM‐Derived Cells

GBM tissue samples (*n* = 6), categorized based on O6‐methylguanine‐DNA methyltransferase (MGMT) promoter methylation status (three methylated, three unmethylated), were surgically collected from patients at CHA Bundang Medical Center.

The obtained tissues were finely minced and enzymatically digested for 30 minutes at 37°C using collagenase D (Roche Diagnostics, Basel, Switzerland) and DNase I (Roche Diagnostics). Following digestion, cells were rinsed in phosphate‐buffered saline (PBS; Thermo Fisher Scientific) and counted using a hemocytometer (Thermo Fisher Scientific).

The isolated cells were then plated at a density of 1.0 × 10^6^ cells/mL in DMEM (Thermo Fisher Scientific) supplemented with 12.5% FBS, 1% penicillin‐streptomycin, 50 ng/mL epidermal growth factor (EGF; Thermo Fisher Scientific), and 50 ng/µL fibroblast growth factor (FGF; PeproTech, Cranbury, NJ, USA). Cells were maintained at 37°C in a humidified incubator containing 5% CO_2_.

### 5.4 Immunofluorescence Analysis

Paraffin‐embedded tissue blocks were sectioned at a thickness of 4 µm. To permeabilize the samples, sections were incubated with 0.5% Triton X‐100 (T8787, Sigma‐Aldrich, St. Louis, MO) for 10 min at room temperature (25°C). Following permeabilization, sections were blocked with CAS‐Block Histochemical Reagent (008120, Thermo Fisher Scientific, Waltham, MA, USA) for 30 min at 25°C. Tissue sections or cells were incubated overnight (16 h) at 4°C with primary antibodies: polyclonal anti‐Fra1 (human FOSL1; reactive to human and mouse) antibody (PA5‐76185, Thermo Fisher Scientific) diluted 1:100, and monoclonal mouse anti‐GFAP antibody (#3670, Cell Signaling Technology) diluted 1:100. After washing, the samples were treated with secondary antibodies for 2 h at 25°C, including goat anti‐rabbit IgG (H+L) Alexa Fluor 488 (A11008, Thermo Fisher Scientific), donkey anti‐rabbit IgG H&L PE (ab7007, Abcam), and goat anti‐mouse IgG H&L Texas Red (ab6787, Abcam), each at a 1:100 dilution. Nuclear staining and mounting were performed using Fluoroshield containing DAPI (F6057, Sigma‐Aldrich). Imaging was conducted using an EVOS M5000 Imaging System (AMF5000, Thermo Fisher Scientific) and an Axio Scan.Z1 slide scanner (Carl Zeiss AG, Jena, Germany) following the manufacturers’ protocols. Quantitative image analysis was performed with ImageJ software version 1.52a (Bethesda, MD, USA). All assessments were carried out under blinded conditions by two independent observers, with five images analyzed per subject within defined regions of interest (ROIs). Mean fluorescence intensity (MFI) was calculated separately for each fluorescence channel (green, red, and blue) in the ROI images using ImageJ. Final MFI values were obtained by subtracting background fluorescence and analyzed individually for each fluorescent channel.

### 5.5 Transfection

To investigate the role of FOSL1 in glioma cells, negative control (NC) and siRNA targeting *FRA1* (si‐FRA1) were employed. The sequences for the FOSL1‐specific siRNAs were CAGCUUUGAGAAGCCUUCA = tt (1‐AS) and UGAAGGCUUCUCAAAGCUG = tt (1‐AA). U87MG cells were seeded into six‐well plates at a density of 6 × 10^5^ cells per well and allowed to adhere for 20–24 h. Approximately 2 h prior to transfection, cells reaching 80%–90% confluence were cultured in fresh serum‐free medium supplemented with antibiotics. Transfection was carried out using Lipofectamine RNAiMAX (13778150, Thermo Fisher Scientific) following the protocol provided by the manufacturer.

### 5.6 qRT‐PCR

U87MG cells, following siRNA transfection, were treated for 48 hours with either TMZ or 0.1% (v/v) dimethyl sulfoxide as a control. After the treatment period, total RNA was isolated using the RNeasy Kit (Qiagen, Hilden, Germany), following the manufacturer's instruction. The concentration, yield, and purity of the extracted RNA were assessed using a NanoDrop spectrophotometer (Thermo Fisher Scientific). High‐quality RNA (2 µg) was reverse transcribed into complementary DNA (cDNA) using the iScript Select cDNA Synthesis Kit (Bio‐Rad, Hercules, CA, USA) with oligo(dT) primers. Quantitative PCR was then performed using the CFX96 Touch Real‐Time PCR Detection System (Bio‐Rad). *FRA1*‐specific primers (Bioneer, Daejeon, South Korea; F: 5‐AGTGGATGGTACAGCCTCATT‐3 R: 5‐CGGGCTGATCTGTTCACAAG‐3), 100 ng cDNA, and SYBR Green Supermix (Bio‐Rad). The relative expression levels were calculated using the ΔΔCt method, with GAPDH (Forward: 5′‐CCTCCAAGGAGTAAGACCCC‐3′, Reverse: 5′‐AGGGGTCTACATGGCAACTG‐3′) serving as the internal control.

### 5.6 Cell Cycle

NC and siRNA‐treated cells (5 × 10^5^ cells/mL) were collected and incubated with Nuclear Green CCS1 dye (#ab112116, Abcam) in 0.5 mL of Fluorometric‐Green cell cycle buffer. The cells were maintained at 37°C for 1 h during staining. Following incubation, cells were washed three times using growth medium supplemented with serum. The stained cells were then resuspended in 0.5 mL of the same assay buffer and subjected to flow cytometric analysis using a CytoFLEX II flow cytometer (Beckman Coulter Inc., Brea, CA, USA). Cell cycle distribution was analyzed using FlowJo software version 10 (FlowJo LLC, Ashland, OR, USA), applying the Watson pragmatic modeling algorithm.

### 5.7 Flow Cytometry

Flow cytometry was employed to assess the expression of FOSL1, MGMT, and proteins related to the cell cycle, stemness, and IL‐6 signaling in U87MG and patient‐derived GBM cells. Prior to staining, Fc receptors were blocked using a human Fc blocking reagent (#564219, BD Biosciences, San Jose, CA). Surface molecules such as CD133 (17‐1338‐42; Thermo Fisher Scientific) and IL‐6R (PA5‐102425; Thermo Fisher Scientific) were stained using specific antibody. For intracellular protein analysis, U87MG cells and patient‐derived GBM cells were collected and incubated in permeabilization buffer (#554722; BD Biosciences) at 4°C for 20 min. After washing with Perm/Wash Buffer, cells were stained at 4°C for 30 min using the following primary antibodies: anti‐FOSL1 (#PA5‐76185; Thermo Fisher Scientific), CDK2 (#LS‐C351983; LSBio), CDK4 (#LS‐C99873; LSBio), cyclin D (#LS‐B4507; LSBio), cyclin E (#32‐1600; Thermo Fisher Scientific), Oct4 (#ab184665; Abcam), Nanog (#ab109250; Abcam), Sox2 (#ab171380; Abcam), MGMT (#MA5‐13506; Thermo Fisher Scientific), IL‐6 (#M620; Thermo Fisher Scientific), STAT3 (#710077; Thermo Fisher Scientific), phospho‐STAT3 Ser727 (#PA5‐17876; Thermo Fisher Scientific), phospho‐STAT3 Tyr705 (#MA5‐15193; Thermo Fisher Scientific), and intracellular ROS (#ab113851; Abcam). After washing step using the Perm/Wash buffer (BD Biosciences), appropriate secondary antibodies were stained for 30 min at 4°C: Goat anti‐Rabbit IgG Alexa 488 (#A11008; Thermo Fisher Scientific), Rat anti‐Mouse PerCP‐eFluor 710 (#46‐4015‐82; Thermo Fisher Scientific), Donkey anti‐Goat APC (#F0108; R&D System, Minneapolis, MN), Goat anti‐Mouse Alexa 488 (#A2112; Thermo Fisher Scientific), and Goat anti‐Rabbit Alexa 647 (#A21244; Thermo Fisher Scientific). All flow cytometry analyses were conducted using the CytoFLEX II system (Beckman Coulter Inc.), and data interpretation was performed using FlowJo software version 10 (FlowJo LLC).

### 5.8 WST‐8 Reduction

The viability of U87MG cells were evaluated using the WST‐8 assay kit (Cellomax, Yongin, South Korea), following the manufacturer's instructions. In brief, GBM cells were seeded into 96‐well plates at a density of 5 × 10^3^ or 1 × 10^4^ cells per well in 100 µL of complete culture medium. After overnight incubation at 37°C with 5% CO_2_ to facilitate adherence, the cells were exposed to either experimental treatments or vehicle controls. Subsequently, 10 µL of WST‐8 reagent was added to each well, and the plates were further incubated at 37°C for 2 or 4 h to allow the generation of formazan, which reflects the number of viable cells. Absorbance at 450 nm was measured using a microplate reader, with background correction performed using a reference wavelength of 600 nm. Cell viability was expressed as a percentage relative to untreated controls, calculated using the following formula:

$$\mathrm{Cell}\;\mathrm{viability}\;\left(\%\right)=100\times \left(\frac{A_{\mathrm{sample}}-A_{\mathrm{blank}}}{A_{\mathrm{control}}-A_{\mathrm{blank}}}\right)$$

Here, A_sample_ indicates the absorbance from wells containing treated cells, A_control_ refers to untreated cell wells, and A_blank_ is the absorbance of the wells containing only medium and reagent (no cells). All experiments were performed in triplicate, and results are reported as mean ± standard deviation.

### 5.9 Colony Formation

NC‐ and si‐*FRA1*‐transfected U87MG cells were plated into six‐well dishes at a density of 1 × 10^4^ cells per well. The cells were then cultured for 7 days at 37°C in a humidified incubator with 5% CO_2_. Following incubation, colonies were fixed and stained with 0.01% crystal violet (#V5265, Sigma‐Aldrich) dissolved in 10% ethanol at room temperature (25°C) for 20 min. The resulting colony formation was visualized and quantified using ImageJ software (version 1.52a, NIH, Bethesda, MD, USA).

### 5.10 Wound Healing Assay

To assess the migratory capacity of U87MG cells, a 35‐mm‐dish equipped with a two‐well culture insert (ibid GmbH, Münster, Germany) was utilized. U87MG cells transfected with either NC or si‐FRA1 were seeded into the inserts and cultured for 24 h to allow the formation of a confluent monolayer. After incubation, the inserts were carefully removed, and fresh medium was added to each well. Cell migration into the cell‐free gap was monitored by capturing images at 0, 6, and 24 h using a microscope. The remaining wound area was analyzed and quantified using ImageJ software (version 1.52a, NIH).

### 5.11 Total RNA Sequencing

To explore the overall transcriptome landscapes, RNA sequencing of GBM tissues was performed (*n* = 53). Total RNA of GBM patients was isolated using the RNeasy Kit (Qiagen), following the manufacturer's protocol. RNA concentration and purity were determined using 1 µL of RNA analyzed with the NanoDrop8000 spectrophotometer, while RNA integrity was evaluated via the Agilent 2100 Bioanalyzer, yielding RNA Integrity Number (RIN) scores. Library construction for total RNA sequencing was performed using the TruSeq Stranded Total RNA Library Prep Kit (Illumina), in line with the supplier's recommendations. For this process, 400 ng of total RNA was first subjected to ribosomal RNA removal using the Ribo‐zero Human/Mouse/Rat Kit (Illumina) with biotin‐labeled probes specific to rRNA. The remaining RNA was fragmented using heat and divalent cations, and reverse transcription was conducted with random primers to synthesize first‐strand cDNA. This was followed by the synthesis of the second strand using RNase H and DNA polymerase I. The resulting double‐stranded cDNA fragments were then end‐repaired, adenylated at the 3' end, ligated to Illumina adapters, and enriched through PCR amplification. The quality of the resulting cDNA libraries was assessed using the Agilent Tapestation system. Quantification of the libraries was performed via qPCR using the KAPA SYBR FAST qPCR Master Mix (Kapa Biosystems). Indexed libraries were pooled at equal molar concentrations and sequenced using the Illumina NovaSeq 6000 platform with a 2 × 100 bp paired‐end configuration. Raw and processed sequencing data were uploaded to the NCBI Gene Expression Omnibus (GEO) under accession number GSE255626.

### 5.12 Glioma Omics Data

Drug sensitivity and RNA sequencing expression data from cancer cell lines in the Cancer Dependency Map (https://depmap.org/portal/) were used to analyze the correlation between gene expression and drug sensitivity in IDH wild‐type cell lines. Drug sensitivity data (PRISM Repurposing Primary Screen, 19Q3) and RNA sequencing data (21Q4) were used. The analysis also included clinical, survival, and RNA‐seq data obtained using the samples from patients with GBM, specifically from TCGA (https://www.cancer.gov/tcga), Chinese Glioma Genome Atlas (CCGA; https://www.cgga.org.cn/), and CHA databases. Data from TCGA and CGGA, including RNA sequencing, clinical, and survival data, were downloaded from GlioVis (http://gliovis.bioinfo.cnio.es//). Only IDH wild‐type samples were analyzed: 220 samples from the TCGA GBMLGG dataset, 225 samples from CGGA, and 52 samples from CHA. Glioma expression subtypes (mesenchymal, classical, and proneural) were classified based on Wang et al. using GBM classification tools from GlioVis. RNA sequencing data of 52 patients were obtained from medical records at CHA. Additionally, 80 samples were analyzed using clinical, RNA sequencing (PRJNA1051047), global proteome (PDC000514), and phosphoproteome (PDC000515) data from Kim et al. to integrate transcriptomic and proteomic data [45].

### 5.13 GSEA and ssGSEA

GSEA was conducted to identify TMZ resistance‐associated terms. First, a co‐expressed transcription factor analysis was performed to determine which transcription factors correlated with TMZ resistance in glioma cells utilizing the human TF ARCHS4 co‐expression database (https://maayanlab.cloud/archs4/) with ShinyGO v. 0.741. Next, we inferred biological processes associated with TMZ resistance through a correlation analysis between the mRNA expression of all genes and TMZ sensitivity data from the DepMap database. Genes with correlation coefficients greater than 0.4 were extracted from GSEA using the Gene Ontology biological process and Hallmark curated databases. Finally, each group was divided using NMF clustering and scored with ssGSEA based on the Hallmark curated database.

### 5.14 NMF Clustering and Bayesian Compound Covariate Prediction

NMF clustering was performed to cluster TCGA, CGGA, and CHA samples based on genes that were moderately positively correlated with TMZ resistance using the NMF Consensus module from GenePattern (https://www.genepattern.org/) with default settings. To ensure consistency, marker genes were extracted from the two groups identified by NMF clustering in the TCGA cohort, and differentially expressed genes were determined using a *t*‐test. Approximately 600 genes were used to perform Bayesian compound covariate (BCC) predictions in the other three cohorts. BCC prediction was conducted with the classpredict package (v. 0.2) using default parameters. Survival analysis was performed using Kaplan–Meier curves to compare prognosis between high and low expression of FOSL1, as well as between groups defined by NMF clustering. The analysis was conducted using the Survival package (v3.2) (https://cran.r‐project.org/web/packages/survival/index.html) and visualized with the Kaplan–Meier method and log‐rank test using the survminer package (v. 0.4.9).

### 5.15 Single‐Cell RNA Sequencing Analysis

Single‐cell RNA sequencing analysis was performed using the Seurat V5 package with approximately 2 × 10^4^ cells from the GSE256493 dataset. Before formal analysis, cells were classified as aneuploid or diploid based on copy number alterations using the CopyKat package (v. 1.1.0). The parameters for the number of genes in the chromosome, window size, and segment size were set to 5, 25, and 0.1, respectively. Cells with fewer than 200 or more than 5500 features, or those with a mitochondrial gene percentage greater than 5%, were filtered out. The remaining data were processed using default parameters. The FindNeighbors and FindClusters functions were applied with 20 principal components and a resolution of 0.25, respectively. Dimensionality reduction and visualization were performed using t‐distributed stochastic neighbor embedding (t‐SNE), resulting in the identification of 11 distinct clusters characterized by established cell‐type markers. Single‐cell enrichment analysis was conducted using the escape package (v. 1.8.0) and visualized with DittoSeq (v. 1.16.0) using the Hallmark databases for enrichment analysis.

### 5.16 In Vivo Studies

In vivo experiments were conducted using 6‐week‐old male BALB/c athymic nude mice weighing 18–20 g (Orient Bio Inc., Seongnam, South Korea). All animal procedures were performed in accordance with the Guidelines for the Care and Use of Laboratory Animals of the Institute of Laboratory Animal Center, Daegu‐Gyeongbuk Medical Innovation Foundation. The animal studies were approved by the Institutional Review Board on the Ethics of Animal Experiments of the Daegu‐Gyeongbuk Medical Innovation Foundation (IACUC approval number: KMEDI‐23091301‐00).

For the first in vivo study evaluating combination therapy in a U87MG/Luc orthotopic brain tumor model, 1 × 10^6^ U87GM/luc cells suspended in 3 µL of sterile PBS were injected into the right frontal hemisphere of the animals using a stereotactic fixation device (Stoelting, Wood Dale, IL, USA). The implants were placed 2 mm from the midline, 3 mm anterior to the bregma, and 3 mm deep. Cells were injected over 120–180 s. Tumor progression was monitored using bioluminescence imaging (BLI). When the bioluminescent signals stabilized, tumor‐bearing mice were divided into four groups: Group 1: vehicle; Group 2: 5 mg/kg TMZ; Group 3: 5 mg/kg VEM; and Group 4: 5 mg/kg TMZ + 5 mg/kg VEM. TMZ and VEM were dissolved in a 0.5% methylcellulose solution and administered by oral gavage once daily. Body weight and survival were measured at designated times.

For the second in vivo study employing a U87MG/Luc subcutaneous xenograft model, mice were subcutaneously injected with 2 × 10^6^ U87MG/luc cells. Tumor‐bearing mice were divided into four groups as follows: Group 1: vehicle, Group 2: 1 mg/kg TMZ, Group 3: 5 mg/kg VEM, and Group 4: 1 mg/kg TMZ + 5 mg/kg VEM. TMZ and VEM were dissolved in a 0.5% methylcellulose solution and administered by oral gavage once daily. Tumor size was measured with calipers at the indicated time points, and tumor volume (mm^3^) was calculated using the following formula: Tumor volume (mm^3^) = *d* ^2^ × *D*/2, where *d* and *D* are the shortest and longest diameters (mm), respectively. Excised tumors were dissected and fixed in 10% formalin for further experiments. Body weight and survival rates were also measured at designated time points.

For in vivo BLI, tumor‐bearing mice received D‐luciferin (PerkinElmer, Waltham, MA, USA) via intraperitoneal injection, and BLI was performed 10 min after substrate injection using the IVIS SPECTRUM (PerkinElmer). All mice were anesthetized with 1%–2% isoflurane gas during imaging. Grayscale photographic images and bioluminescent color images were superimposed using LIVINGIMAGE (version 2.12; PerkinElmer) and IGOR Image Analysis FX software (WaveMetrics, Lake Oswego, OR, USA). BLI signals were expressed as units of photons per cm^2^ per second per steradian (P/cm^2^/s/sr).

### 5.17 TUNEL Assay

To assess apoptosis within GBM mouse tissues (intracranial tumor injection model) treated with TMZ or VEM, 4‐µm‐thick sections of FFPE mouse GBM specimens were prepared. Tissue slices were permeabilized using 0.1% Triton X‐100 (w/v) and subsequently incubated with a TUNEL detection reagent (Merck GmbH, Darmstadt, Germany) to label fragmented DNA. The sections were counterstained with DAPI for 30 min to visualize nuclei. Apoptotic cells were evaluated using a slide scanner (Axio Scan.Z1, Carl Zeiss), and quantification was performed using ImageJ software (version 1.52a; NIH).

### 5.18 Statistical Analysis

All data are expressed as means ± standard deviation and statistical significance was determined using an unpaired Student's *t*‐test, paired *t*‐test, and one‐way ANOVA in GraphPad Prism 8 (La Jolla, CA, USA). *p* values < 0.05 were considered statistically significant.

## Author Contributions

Conceptualization: J. M. Sim, J. M. Park, and J. J. Lim. Methodology: J. M. Sim, J. M. Park, J. H. Heo, J. E. Lee, Y. H. Jeon, S. H. Choi, and H. J. Kim. Software: J. M. Park. Validation: J. M. Sim, J. M. Park, Y. J. Kim, D. K. Kim, S. J. Hwang, Y. H. Jeon, and J. E. Lee. Visualization: J. M. Sim and J. M. Park. Formal analysis: J. M. Sim and J. M. Park: Investigation: J. M. Sim, J. M. Park, Y. H. Jeon, J. E. Lee, J. H. Kim, J. H. Yoo, and J. H. Moon. Resources: J. H. Heo, J. H. Yoo, J. H. Moon, K. G. Cho, K. S. Sung, and J. J. Lim. Data curation: J. M. Sim, J. M. Park, K. S. Sung, and J. J. Lim. Writing – original draft preparation: J. M. Sim and J. M. Park. Writing – review and editing: J. M. Sim, J. M. Park, J. H. Heo, J. S. Moon, K. S. Sung, and J. J. Lim. Supervision: J. S. Moon, K. S. Sung, and J. J. Lim. Project administration: J. S. Moon, K. S. Sung, and J. J. Lim. All authors have read and approved the final manuscript.

## Funding

This research was supported by The Ministry of Science, Technology, and Information of the Republic of Korea (Grant Nos. RS‐2024‐00357185 RS‐2024‐00353561, and 2022R1F1A1067203).

## Ethics Statement

This human subject study was conducted in accordance with the Declaration of Helsinki, with its protocol approved by the Institutional Review Board of the Bundang CHA Medical Center (IRB number: CHAMC2021‐01‐024). All animal procedures were performed in accordance with the Guidelines for the Care and Use of Laboratory Animals of the Institute of Laboratory Animal Center, Daegu‐Gyeongbuk Medical Innovation Foundation. The animal studies were approved by the Institutional Review Board on the Ethics of Animal Experiments of the Daegu‐Gyeongbuk Medical Innovation Foundation (IACUC approval number: KMEDI‐23091301‐00).

## Consent

Informed consent was obtained from all subjects involved in the study.

## Conflicts of Interest

Sojung Hwang conducted the present study while affiliated with Bundang CHA Hospital and CHA University. She is currently employed at GC Cell Corporation. The author declares that there are no financial relationships, partnerships, or commercial interests with this company related to the present study. The authors declare that the animal experiments were performed under a fee‐for‐service contract with the Preclinical Research Center, K‐Medi Hub, paid in full using institutional research funds. Outside this contractual relationship, the authors have no financial, professional, or personal conflicts of interest with K‐Medi Hub. The authors affirm that K‐Medi Hub had no role in the study design; data collection, analysis, or interpretation; manuscript writing; or the decision to publish. The support provided did not affect the outcomes or conclusions of this independent research. The authors commissioned BioArtLab to create the graphical abstract using their own institutional research funding. There are no financial relationships, partnerships, or commercial interests between the authors and BioArtLab related to this work.

## Supporting information



Supporting information

## Data Availability

The authors have nothing to report.
