# Supplementary material for: Inhibition of FOS‐Like Antigen 1 Reduces Chemoresistance to Temozolomide Through Stemness Reprogramming via IL‐6/STAT3Tyr705 Pathway
Source: MedComm (2020). 2026 Jan 18;7(2):e70593. doi: 10.1002/mco2.70593 (PMC12812332; doi:10.1002/mco2.70593)
Supplement: Supplementary file 1 — Supporting information [file MCO2-7-e70593-s001.pdf]

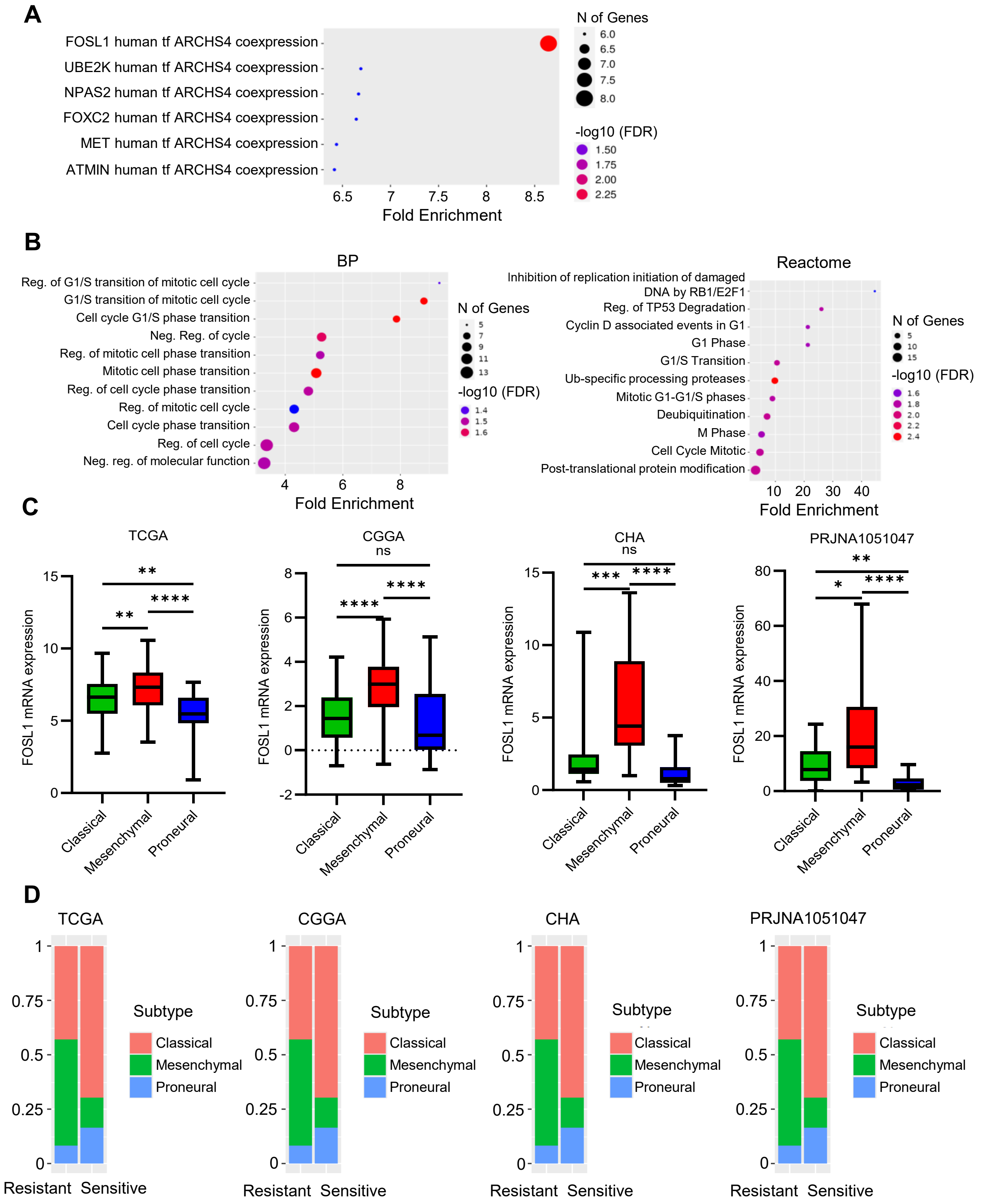

A
FOSL1 human tf ARCHS4 coexpression
UBE2K human tf ARCHS4 coexpression
NPAS2 human tf ARCHS4 coexpression
FOXC2 human tf ARCHS4 coexpression
MET human tf ARCHS4 coexpression
ATMIN human tf ARCHS4 coexpression
Fold Enrichment
N of Genes
-log10 (FDR)
B
BP
Reg. of G1/S transition of mitotic cell cycle
G1/S transition of mitotic cell cycle
Cell cycle G1/S phase transition
Neg. Reg. of cycle
Reg. of mitotic cell phase transition
Mitotic cell phase transition
Reg. of cell cycle phase transition
Reg. of mitotic cell cycle
Cell cycle phase transition
Reg. of cell cycle
Neg. reg. of molecular function
Fold Enrichment
Reactome
Inhibition of replication initiation of damaged DNA by RB1/E2F1
Reg. of TP53 Degradation
Cyclin D associated events in G1
G1 Phase
G1/S Transition
Ub-specific processing proteases
Mitotic G1-G1/S phases
Deubiquitination
M Phase
Cell Cycle Mitotic
Post-translational protein modification
Fold Enrichment
C
TCGA
CGGA
CHA
PRJNA1051047
FOSL1 mRNA expression
Classical
Mesenchymal
Proneural
D
TCGA
CGGA
CHA
PRJNA1051047
Subtype
Classical
Mesenchymal
Proneural
Resistant
Sensitive

Fig. S1. FOSL1 is positive correlated with chemoresistance and mesenchymal signature in GBM. A FOSL1 is a highly co-expressed transcription factor with gene expression and is positively correlated with TMZ resistance. **B** GSEA of genes whose expression was positively correlated with TMZ resistance. **C** Comparing expression of FOSL1 among the three GBM expression subtypes. **D** Comparing and the proportion of expression subtypes between the two groups in four datasets.

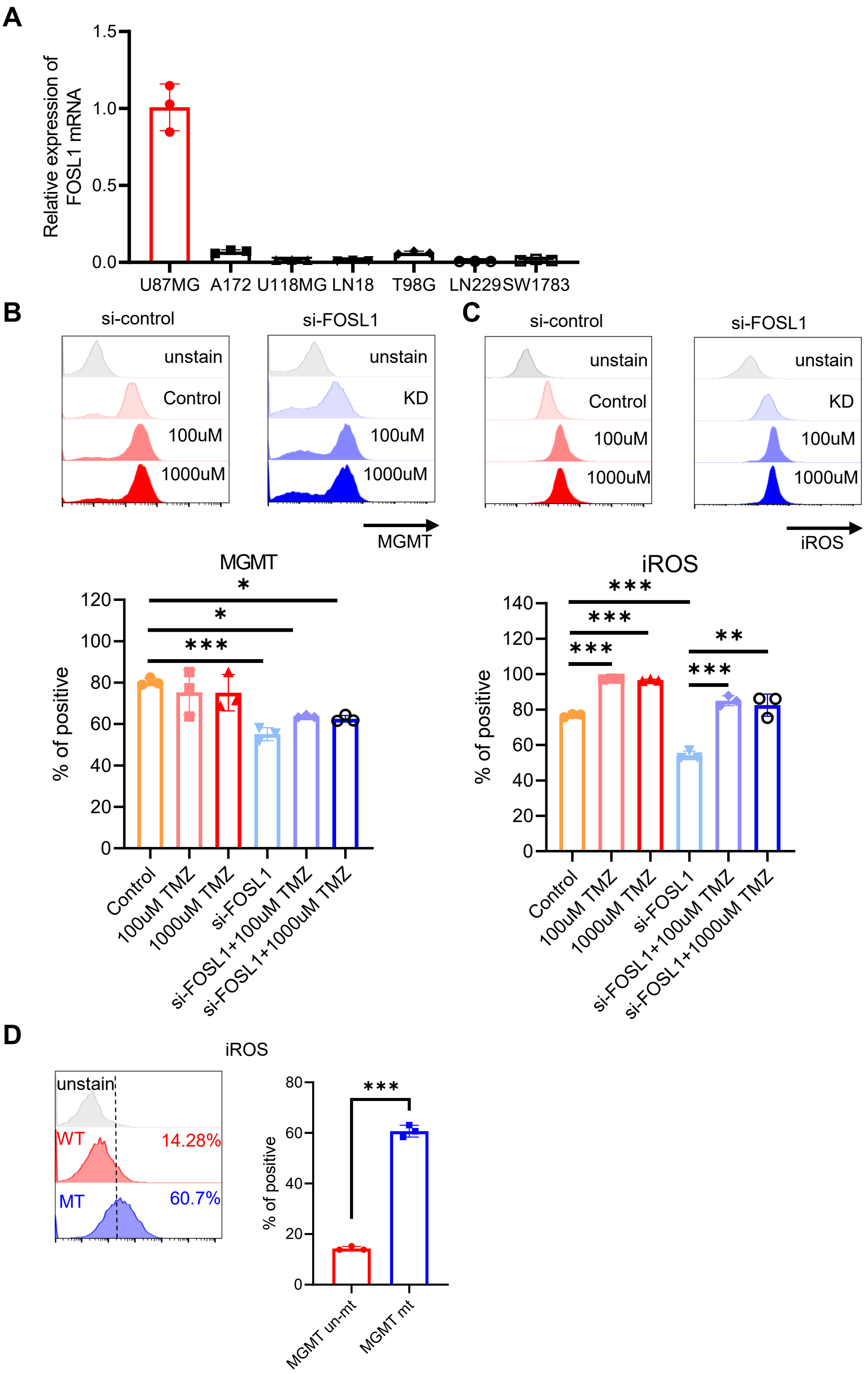
A
Relative expression of FOSL1 mRNA
1.5
1.0
0.5
0.0
U87MG
A172
U118MG
LN18
T98G
LN229
SW1783
B
si-control
si-FOSL1
unstain
Control
100uM
1000uM
KD
MGMT
% of positive
120
100
80
60
40
20
0
*
***
Control
100uM TMZ
1000uM TMZ
si-FOSL1
si-FOSL1+100uM TMZ
si-FOSL1+1000uM TMZ
C
iROS
140
**
D
WT
MT
14.28%
60.7%
MGMT un-mt
MGMT mt

Fig. S2. FOSL1 is associated with MGMT and ROS in GBM cells. **A** cDNA of GBM cell lines analyzed using qPCR to detect *FOSL1* mRNA expression. For relative quantification, *GAPDH* was used as a control gene. **B, C** Representative histograms of MGMT and iROS levels (top panel). To detect MGMT and iROS expression in GBM cells, U87MG cells were pretreated with temozolomide and si-FOSL1. Each protein was analyzed using flow cytometry and quantified using FlowJo V10 (bottom panel) (n = 3). **D** iROS in patients with GBM-derived cells (n = 6) was analyzed by flow cytometry and quantified using FlowJo V10 (bottom panel). *$^{*}p < 0.05$; $^{**}p < 0.005$; $^{***}p < 0.0005$; paired t-test.*

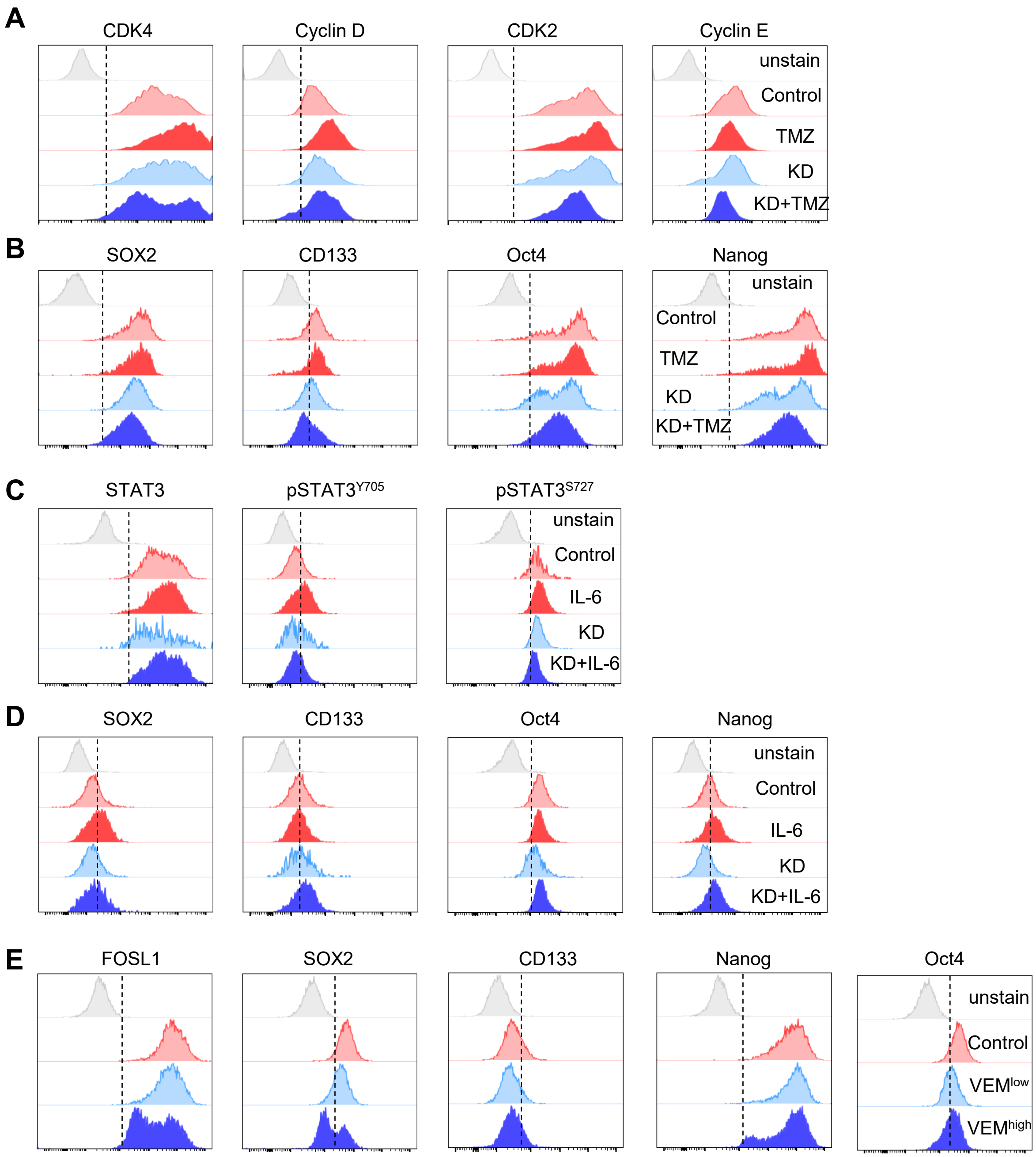
A
CDK4
Cyclin D
CDK2
Cyclin E
unstain
Control
TMZ
KD
KD+TMZ
B
SOX2
CD133
Oct4
Nanog
unstain
Control
TMZ
KD
KD+TMZ
C
STAT3
pSTAT3Y705
pSTAT3S727
unstain
Control
IL-6
KD
KD+IL-6
D
SOX2
CD133
Oct4
Nanog
unstain
Control
IL-6
KD
KD+IL-6
E
FOSL1
SOX2
CD133
Nanog
Oct4
unstain
Control
VEMlow
VEMhigh

Fig. S3. Protein expression patterns of cell cycle, stemness and IL-6 pathway-associated molecules by FOSL1 downregulation in U87MG cells. A-E Representative histograms of FOSL1, cell cycle, stemness and IL-6 pathway-associated molecules in U87MG cells treated with TMZ, si-FOSL1, IL-6 or vemurafenib (VEM). Each protein was analyzed using flow cytometry and quantified using FlowJo V10 (n = 3).

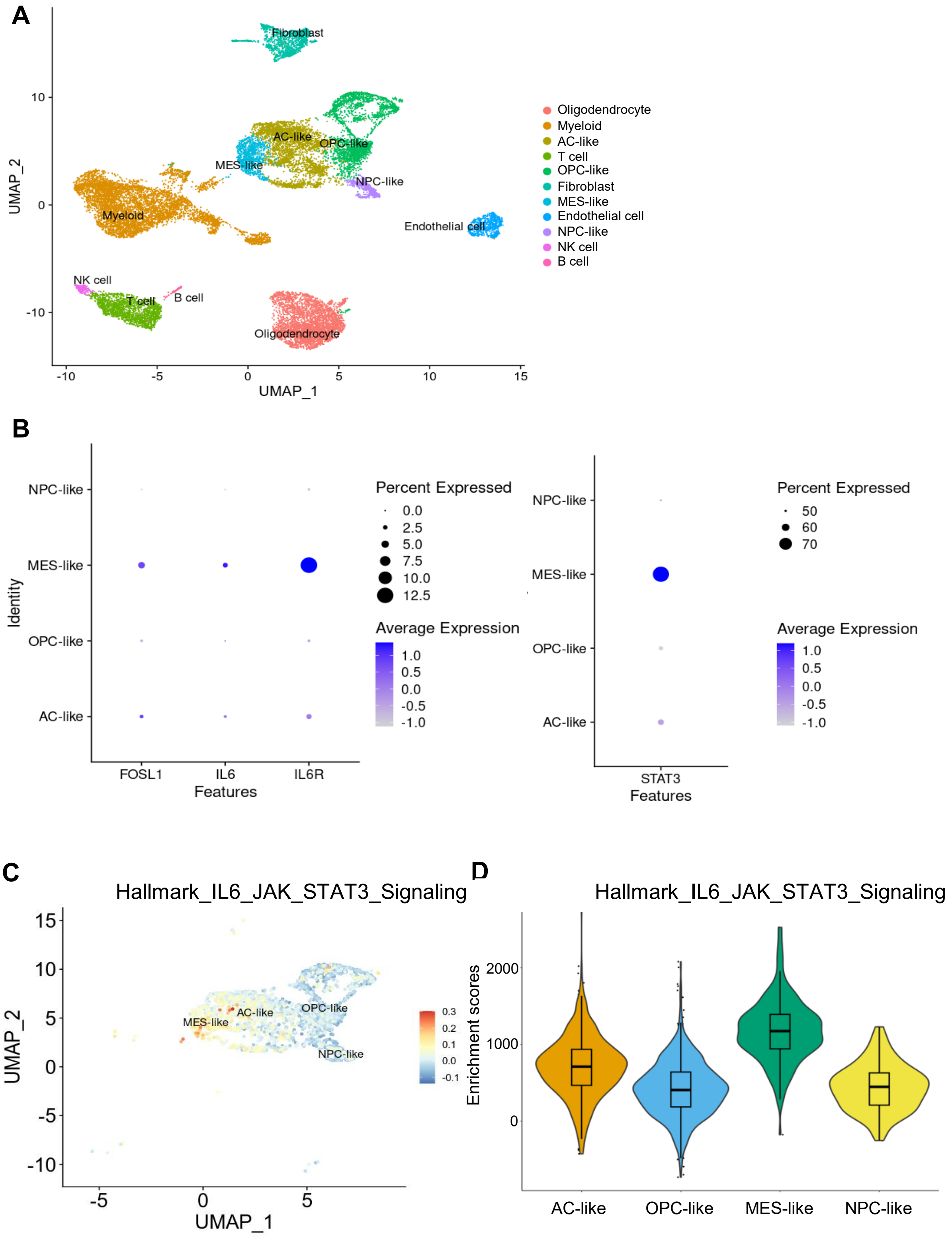

A
Fibroblast
AC-like
OPC-like
MES-like
NPC-like
Myeloid
Endothelial cell
NK cell
T cell
B cell
Oligodendrocyte
UMAP_1
UMAP_2
Oligodendrocyte
Myeloid
AC-like
T cell
OPC-like
Fibroblast
MES-like
Endothelial cell
NPC-like
NK cell
B cell
B
Identity
NPC-like
MES-like
OPC-like
AC-like
FOSL1
IL6
IL6R
Features
Percent Expressed
0.0
2.5
5.0
7.5
10.0
12.5
Average Expression
1.0
0.5
0.0
-0.5
-1.0
STAT3
Percent Expressed
50
60
70
C
Hallmark_IL6_JAK_STAT3_Signaling
D
Hallmark_IL6_JAK_STAT3_Signaling
Enrichment scores
AC-like
OPC-like
MES-like
NPC-like

Fig. S4. Single cell landscape of FOSL1 and IL-6 pathway-associated molecules in GBM. **A** The UMAP plot of single cell RNA-sequencing data. **B** Expression of FOSL1 and IL-6 in aneuploid cells, except for oligodendrocytes, endothelial cells, fibroblasts, and immune cells. **C** Dim plot of single-cell GSEA (scGSEA) of IL-6-JAK-STAT3 signaling pathway in Hallmark. **D** Comparison of scGSEA score between four types of aneuploid cells.

Table S1. Baseline characteristic of patients with GBM subjects.

	TMZ resistant GBM (n=3)	TMZ sensitive GBM (n=3)
Sex	1 Male / 2 Female	3 Male
Age	62.3 ± 4.99	66.3 ± 8.99
IDH1 mutation	Wild type	
Ki-67 (%)	15 ± 4.08	23.3 ± 16.5
MGMT methylation	-	+
